# Combined glutamine, dietary fiber, and oligosaccharide supplementation attenuates fasting-associated intestinal dysbiosis and stress responses in rats

**DOI:** 10.3389/fnut.2026.1933552

**Published:** 2026-09-17

**Authors:** Yuta Murai, Takumi Iwasawa, Satoshi Takeda, Kaho Ohki, Kazunori Kato, Tomoaki Ito

**Affiliations:** 1 Shizuoka Medical Research Center for Disaster, Juntendo University, Shizuoka, Japan; 2 Department of Surgery, Juntendo University Shizuoka Hospital, Shizuoka, Japan; 3 Institute of Life Innovation Studies, Toyo University, Tokyo, Japan; 4 Department of Nutritional Sciences, Graduate School of Health and Sports Sciences, Toyo University, Tokyo, Japan

**Keywords:** autophagy, glutamine dietary fiber and oligosaccharides, gut microbiota, intestinal dysbiosis, intestinal homeostasis, perioperative nutrition, prolonged fasting

## Abstract

**Background:**

Prolonged preoperative fasting is associated with intestinal dysbiosis, impaired mucosal homeostasis, and delayed postoperative recovery. Combined nutritional supplementation with glutamine, dietary fiber, and oligosaccharides has the potential to protect intestinal function during nutritional stress. In this study, we investigated the effects of this nutritional combination in a rat model of prolonged fasting.

**Methods:**

Male Sprague-Dawley rats received oral supplementation with a combination of glutamine, dietary fiber, and oligosaccharides (GFO) or a 15% glucose solution twice daily for 9 days and were subjected to fasting from days 6 to 9. Jejunal morphology, short-chain fatty acid (SCFA) concentrations, intestinal gene expression, and gut microbiota composition were evaluated.

**Results:**

Fasting significantly reduced body weight, jejunal villous height, cecal weight, and the concentrations of acetate, n-butyrate, and n-valerate. Fasting was also associated with exploratory changes in gut microbiota composition and increased the expression of several Toll-like receptor (TLR), barrier, and autophagy-related genes in the jejunum. Supplementation with glutamine, dietary fiber, and oligosaccharides significantly attenuated the fasting-induced reduction in cecal weight and suppressed the upregulation of TLR5, TLR6, TLR8, CLDN1, CLDN3, OCLN, ZO-1, MUC3, ATG5, ATG12, Beclin1, p62, and LC3. Microbiota analysis showed that the relative abundance of sequences tentatively assigned to *Lactobacillus reuteri* in the supplemented group was comparable to that in the control group, whereas sequences tentatively assigned to *Bifidobacterium animalis* were detected predominantly in the supplemented group. The relative abundances of *Desulfovibrio, Bilophila*, and *Mucispirillum schaedleri* in the supplemented group appeared closer to those observed in the control group than to those in the fasting group. Furthermore, supplementation significantly increased propionate levels compared with the control group.

**Conclusions:**

Combined supplementation with glutamine, dietary fiber, and oligosaccharides attenuated several fasting-associated intestinal stress responses and was accompanied by exploratory changes in gut microbiota composition and SCFA profiles. These intestinal effects were accompanied by exploratory changes in gut microbiota composition and SCFA profiles, together with attenuation of fasting-associated changes in intestinal stress-related markers, suggesting that this nutritional strategy may help maintain intestinal homeostasis during prolonged fasting and may have potential applications in perioperative nutritional support.

## 1 Introduction

Preoperative fasting has traditionally been used to reduce the risk of aspiration and anesthesia-related complications. However, contemporary Enhanced Recovery After Surgery (ERAS) protocols recommend minimizing the duration of fasting because prolonged fasting may adversely affect metabolic homeostasis, immune function, and intestinal health, ultimately impacting surgical outcomes and patient recovery (1, 2). Nevertheless, unavoidable fasting remains common in clinical practice. Patients with upper gastrointestinal stenosis, colorectal obstruction, acute gastroenteritis, or postoperative ileus often require prolonged periods without oral intake, highlighting the need for nutritional strategies that can mitigate fasting-associated intestinal dysfunction. Extended preoperative fasting has been shown to exacerbate the catabolic response, promote insulin resistance, and delay postoperative gastrointestinal function recovery, all of which are critical factors in gastrointestinal surgery (3, 4).

Beyond metabolic implications, prolonged fasting also negatively affects host immunity. Several studies have demonstrated that nutrient deprivation impairs immune surveillance and mucosal immune responses, thereby increasing the risk of postoperative infections and delayed wound healing (5–7). These immunosuppressive effects are particularly relevant in gastrointestinal surgeries, where a compromised intestinal barrier can exacerbate microbial translocation and inflammation, leading to complications such as sepsis (8).

The gut microbiota plays a central role in maintaining intestinal homeostasis, regulating mucosal immunity, and supporting the epithelial barrier. However, fasting disrupts microbial diversity and composition, often favoring the expansion of opportunistic pathogens and reducing beneficial commensals (9, 10). These alterations can further compromise gut barrier function and impair postoperative recovery.

In recent years, perioperative nutritional interventions have garnered attention for their potential to mitigate the adverse effects of fasting. Glutamine, a conditionally essential amino acid, has been extensively studied for its role in preserving intestinal epithelial integrity, enhancing tight junction protein expression, and modulating immune cell function (11, 12). Dietary fibers and prebiotic oligosaccharides also contribute significantly to gut health by promoting the growth of beneficial microbiota, producing short-chain fatty acids (SCFAs), and supporting mucosal immunity (13, 14).

Several clinical and preclinical studies have highlighted the efficacy of combined supplementation with glutamine, dietary fiber, and oligosaccharides (GFO) in reducing postoperative complications, shortening the duration of ileus, and accelerating the return of bowel function (15–18). These findings suggest that targeted nutritional strategies can counteract the negative effects of fasting and optimize perioperative care.

Therefore, strategies that can attenuate the adverse effects of fasting on intestinal homeostasis may have important clinical value, particularly in situations where fasting cannot be avoided.

In this study, we investigated the effects of combined supplementation with glutamine, dietary fiber, and oligosaccharides in a rat model of prolonged fasting. Specifically, we evaluated fasting-associated changes in intestinal morphology, cecal microbiota composition, SCFA profiles, and intestinal stress-related markers, including barrier- and autophagy-associated molecules. We aimed to determine whether these parameters were altered concurrently following preventive nutritional supplementation and to provide preliminary insight into their potential relationships during prolonged fasting.

## 2 Materials and methods

### 2.1 Combination of GFO

GFO is a commercially available enteral nutritional supplement containing glutamine, dietary fiber (polydextrose), and lactosucrose as its principal functional ingredients. According to the manufacturer's product information, each 15-g sachet contains approximately 5 g glutamine, 7.5 g polydextrose, and 1.45 g lactosucrose, together with vitamins and minerals. In the present study, GFO was suspended in distilled water immediately before administration and orally administered at a dose of 3 g/kg body weight twice daily. The present study was designed to evaluate the preventive biological effects of combined supplementation with glutamine, dietary fiber, and oligosaccharides when initiated before and continued during fasting. The study was not designed to assess therapeutic or recovery-promoting effects after fasting-induced intestinal injury.

### 2.2 *In vivo* fasting rat model

Male rat (Slc:SD, origin: Charles River Laboratory Inc., total *n* = 24) were purchased from Japan SLC, Inc. (Hamamatsu, Japan). The rat was housed under a 12 h light/12 h dark cycle with free access to food and water and were allowed to acclimate for at least 1 week. The housing chamber temperature was maintained at approximately 23 °C. Rats were randomly assigned to four experimental groups (*n* = 6 per group) as follows:

(1) Control group (Control): animals received a standard diet *ad libitum* and no oral treatment.
(2) Fasting group (Fasting): animals underwent complete food deprivation from days 6 to 9 without oral treatment.
(3) Glucose group (Glucose): animals were orally administered a 15% glucose solution (3 g/kg body weight) twice daily for 9 consecutive days and were fasted from days 6 to 9. The 15% glucose solution was included as a simple carbohydrate comparator during fasting and was not intended to serve as a calorie-, osmolality-, or composition-matched control for the GFO formulation.
(4) GFO group (GFO): animals were orally administered GFO solution (3 g/kg body weight) twice daily for 9 consecutive days and were fasted from days 6 to 9. Supplementation was initiated 5 days before fasting and continued throughout the 3-day fasting period to evaluate its preventive effects against fasting-associated intestinal alterations. No post-fasting treatment or recovery phase was included in the present experimental design.

GFO solution was composed of a mixture of glutamine, dietary fiber, and oligosaccharides, and glucose solution was prepared from D-(+)-glucose (Otsuka Pharmaceutical Factory, Inc., Tokushima, Japan) dissolved in sterile distilled water. Oral administration was performed by gavage twice daily at approximately 10:00 and 18:00 throughout the 9-day treatment period. During the fasting period (days 6–9), animals had free access to water.

On day 9, animals were euthanized under deep isoflurane anesthesia, and blood was collected from the abdominal aorta using a sterile syringe. Serum was obtained by centrifugation at 3,000 rpm for 10 min and stored at −80 °C until analysis. The jejunum and cecum were excised, gently flushed with cold phosphate-buffered saline (PBS), and processed for downstream analysis. Tissue samples were either snap-frozen in liquid nitrogen for molecular assays or fixed in 10% neutral-buffered formalin for histological examination.

The animal study was reviewed and approved by the Animal Care and Use Committee of the Juntendo University School of Medicine (approval number: 2022139).

### 2.3 Histological analysis of the jejunum

To assess jejunal morphology, a segment of the jejunum was collected from each rat, defined as the portion of small intestine located more than 8 cm distal to the pyloric sphincter. The tissue was gently flushed with PBS and immediately fixed in 10% neutral-buffered formalin. Fixed samples were outsourced to Sapporo Pathology Inspection Center Co., Ltd. (Sapporo, Japan) for paraffin embedding, microtome sectioning (4 μm thickness), and hematoxylin and eosin (H&E) staining.

Villous height was measured using a Keyence digital microscope analyzer (Model: BZ-X710). For each rat, 50 villi were randomly selected from well-oriented longitudinal sections, and their heights (from the tip to the crypt base) were measured. The mean villous height was calculated for each animal. A total of six animals per group (*n* = 6) were evaluated.

### 2.4 Gene expression analysis

Total RNA was extracted from frozen jejunal tissue using a μT-12 Beads Crusher (TAITEC Co., Saitama, Japan) and the RNeasy Mini Kit (QIAGEN, Hilden, Germany) according to the manufacturer's instructions. RNA concentration and purity were assessed using a microvolume spectrophotometer DS-11 FX (Thermo Fisher Scientific, Waltham, MA, USA). Complementary DNA (cDNA) was synthesized from 500 ng of total RNA using the ReverTra ACR^®^ qPCR RT Master Mix (TOYOBO Co., Ltd., Osaka, Japan) following the manufacturer's protocol. Quantitative real-time PCR (qPCR) was performed using the Thermal Cycler Dice^®^ Real Time System III (TP900, Takara Bio Inc., Shiga, Japan). Each PCR reaction was conducted in triplicate. Relative mRNA expression levels were calculated using the 2^−Δ*ΔCt*^ method, with Glyceraldehyde-3-Phosphate Dehydrogenase (GAPDH) as the internal control. The sequences of the primers used for each target gene are listed in Supplementary Table 1.

### 2.5 SCFA analysis

SCFA concentrations in cecal contents were measured to assess microbial metabolic activity. Cecal samples were collected immediately after euthanasia and stored at −80 °C until analysis. SCFA profiling was outsourced to Techno Suruga Laboratory Co., Ltd. (Shizuoka, Japan).

The following SCFAs were quantified: acetate, propionate, n-butyrate, iso-butyrate, n-valerate, and iso-valerate. A total of six animals per group (*n* = 6) were analyzed.

### 2.6 Cecal microbiota analysis by 16S rDNA (ribosomal DNA) amplicon sequencing

Cecal microbiota composition was analyzed by 16S rRNA gene amplicon sequencing using samples collected from the Control, Fasting, GFO, and Glucose groups described above. Cecal contents were aseptically collected immediately after euthanasia, stored at −80 °C, and submitted to Techno Suruga Laboratory Co., Ltd. (Shizuoka, Japan) for analysis. Three biological replicates per group (*n* = 3) were analyzed.

Sequencing was performed using the Illumina MiSeq platform. Primer sequences were removed using Cutadapt (v3.5), and paired-end reads were merged using fastq-join (v1.3.1). Quality filtering and amplicon sequence variant inference were performed in QIIME 2 (v2020.6) using DADA2 (v1.10.0). Taxonomic classification was conducted against the SILVA reference database (v138). Because species-level identification based on short 16S rRNA gene amplicons has limited taxonomic resolution, species-level assignments were considered tentative. Alpha diversity was assessed using the Chao1, Shannon, and Simpson indices. Pairwise comparisons were performed using nonparametric tests, and the resulting *p*-values were adjusted using the Benjamini–Hochberg false discovery rate method. Beta diversity was calculated using Bray–Curtis distances and visualized by three-dimensional principal coordinate analysis. Pairwise differences in community composition were evaluated by analysis of similarities (ANOSIM) using 999 permutations, followed by false discovery rate correction. Data visualization was conducted in R (v3.4.1) using qiime2R (v0.99.13) and tidyverse (v1.2.1).

### 2.7 Western blot analysis

Jejunal tissue proteins were extracted and subjected to SDS-PAGE followed by transfer to a PVDF membrane. Membranes were incubated with primary antibodies against ATG12 (Cell Signaling Technology, Danvers, MA, USA; #4180), ATG5 (Cell Signaling Technology; #12994), and β-actin (Cell Signaling Technology; #4967), followed by anti-rabbit IgG HRP-linked (Cell Signaling Technology; #7074). Signals were developed using Pierce™ ECL Western Blotting Substrate (Thermo Fisher Scientific; 32209) and imaged with iBright CL1000 (Thermo Fisher Scientific). Band intensities were quantified using ImageJ v1.53e (NIH, Bethesda, MD, USA) software and normalized to β-actin. Relative expression levels were calculated with the Control group set to 1. Three biological replicates per group were analyzed.

### 2.8 Statistical analysis

Data are presented as mean ± standard deviation (SD) unless otherwise stated. For comparisons between two groups, Welch's *t*-test (two-tailed) was used. For comparisons among multiple groups, one-way ANOVA was performed, followed by Dunnett's multiple comparisons test. For the gene-expression analyses, *p*-values obtained for the 24 genes examined were additionally adjusted for multiple testing using the Benjamini–Hochberg false discovery rate (FDR) procedure separately for each prespecified group comparison. An FDR-adjusted *q* < 0.05 was considered statistically significant. For longitudinal body weight data, a two-way repeated-measures ANOVA (group × time) was used. When variance heterogeneity was detected, Welch's ANOVA was applied with appropriate *post hoc* testing. *p* < 0.05 was considered significant. Statistical analyses and graph generation were performed using R v3.4.1 and Microsoft Excel v16.105.1 (Microsoft Corporation, Redmond, WA, USA).

## 3 Results

### 3.1 Effects of GFO administration on body weight, jejunal villous morphology, and cecal weight during fasting

An overview of the experimental design for the fasting rat model is shown in Figure 1A. Changes in body weight during the experimental period are presented in Figure 1B. Compared with the Control group, body weight was decreased in the Fasting, GFO, and Glucose groups. However, no significant differences in body weight were observed among the Fasting, GFO, and Glucose groups. Representative Hematoxylin-Eosin (H&E) stained images of the jejunal villi are shown in Figure 1C. Villous height was measured as indicated by the green lines. Quantitative analysis revealed that jejunal villous height was significantly reduced in the Fasting group compared with the Control group (Figure 1D). Although the GFO group showed a tendency toward reduced villous height compared with the Control group, the difference was not statistically significant. In addition, no significant difference was observed between the Fasting and GFO groups. To evaluate intestinal atrophy associated with fasting, cecal weight normalized to body weight was measured (Figure 1E). Cecal weight was significantly decreased in the Fasting group compared with the Control group. Notably, GFO administration significantly attenuated the fasting-induced reduction in cecal weight. These findings indicate that fasting induces body weight loss, jejunal villous shortening, and a reduction in relative cecal weight. GFO administration significantly attenuated the reduction in relative cecal weight, whereas no significant preservation of jejunal villous height was observed.

**Figure 1:**
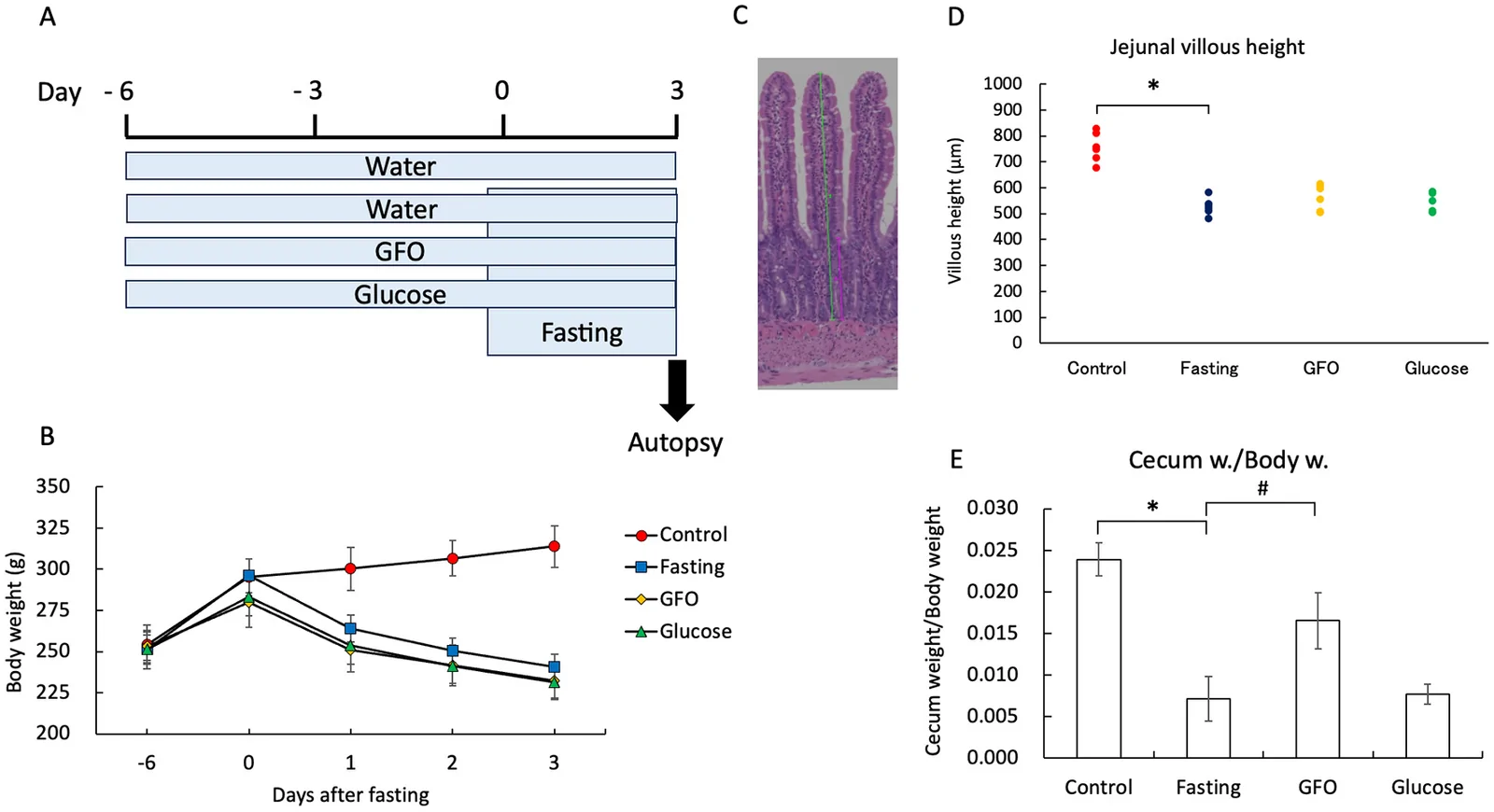
Effects of GFO administration on body weight, jejunal villous morphology, and cecal weight in fasting rats. **(A)** Schematic representation of the experimental protocol for the fasting rat model. Rats received oral administration of GFO solution or a 15% glucose solution twice daily from day −6 to day 3, with the initiation of fasting defined as day 0. Fasting was performed from day 0 to day 3. **(B)** Changes in body weight during the experimental period. Body weight was reduced in the Fasting, GFO, and Glucose groups compared with the Control group. No significant differences were observed among the fasting groups. **(C)** Representative Hematoxylin-Eosin (H&E) stained images of jejunal villi. Green lines indicate villous height measurements. **(D)** Quantification of jejunal villous height. Fifty villi per animal were measured, and the mean value was calculated for each rat. Villous height was significantly decreased in the Fasting group compared with the Control group. Although villous height tended to decrease in the GFO group, no significant difference was observed compared with the Control group or the Fasting group. **(E)** Cecal weight normalized to body weight. Fasting significantly reduced cecal weight compared with the control group, whereas GFO administration significantly attenuated this reduction. Data are presented as mean ± SD. *p < 0.05 vs. the control group; ^#^*p* < 0.05 vs. the fasting group.

### 3.2 GFO suppressed fasting-induced upregulation of Toll-like receptor (TLR), barrier, and autophagy related genes in the jejunum

To investigate the effects of fasting and GFO administration on intestinal immune signaling, barrier-associated molecules, and autophagy-related pathways, mRNA (messenger RNA) expression in the jejunum was analyzed by Quantitative Reverse Transcription PCR (RT-qPCR). As shown in Figure 2A, fasting induced the expression of several Toll-like receptor (TLR)-related genes. In particular, after false discovery rate (FDR) correction, the expression levels of TLR5 and TLR8 were significantly increased in the Fasting group compared with the Control group (Figure 2A). GFO administration significantly attenuated the fasting-associated changes in TLR5, TLR6, and TLR8 expression compared with the Fasting group after FDR correction. Although TLR4 expression was lower in the GFO group than in the Fasting group, this difference did not remain statistically significant after FDR correction. Next, the expression of intestinal barrier- and mucin-related genes was evaluated (Figure 2B). After FDR correction, fasting significantly increased the expression of CLDN1, CLDN3, OCLN, ZO-1, and MUC3 compared with the Control group. GFO administration significantly attenuated the fasting-associated increases in all five of these genes. Although Ambra1 expression showed differences in the original analysis, these differences did not remain statistically significant after FDR correction. No significant differences were observed in CLDN4, IFN-γ, TNF-α, or MUC2 after FDR correction. Furthermore, fasting significantly increased the expression of the autophagy-related genes ATG5, ATG12, Beclin1, p62, and LC3 compared with the Control group after FDR correction (Figure 2C). GFO administration significantly attenuated the fasting-associated increases in all five autophagy-related genes after FDR correction. Consistent with the mRNA findings, western blot analysis showed significantly higher ATG12 and ATG5 protein expression in the Fasting group than in the Control group, whereas both proteins were significantly lower in the GFO group than in the Fasting group (Supplementary Figure S1).

**Figure 2:**
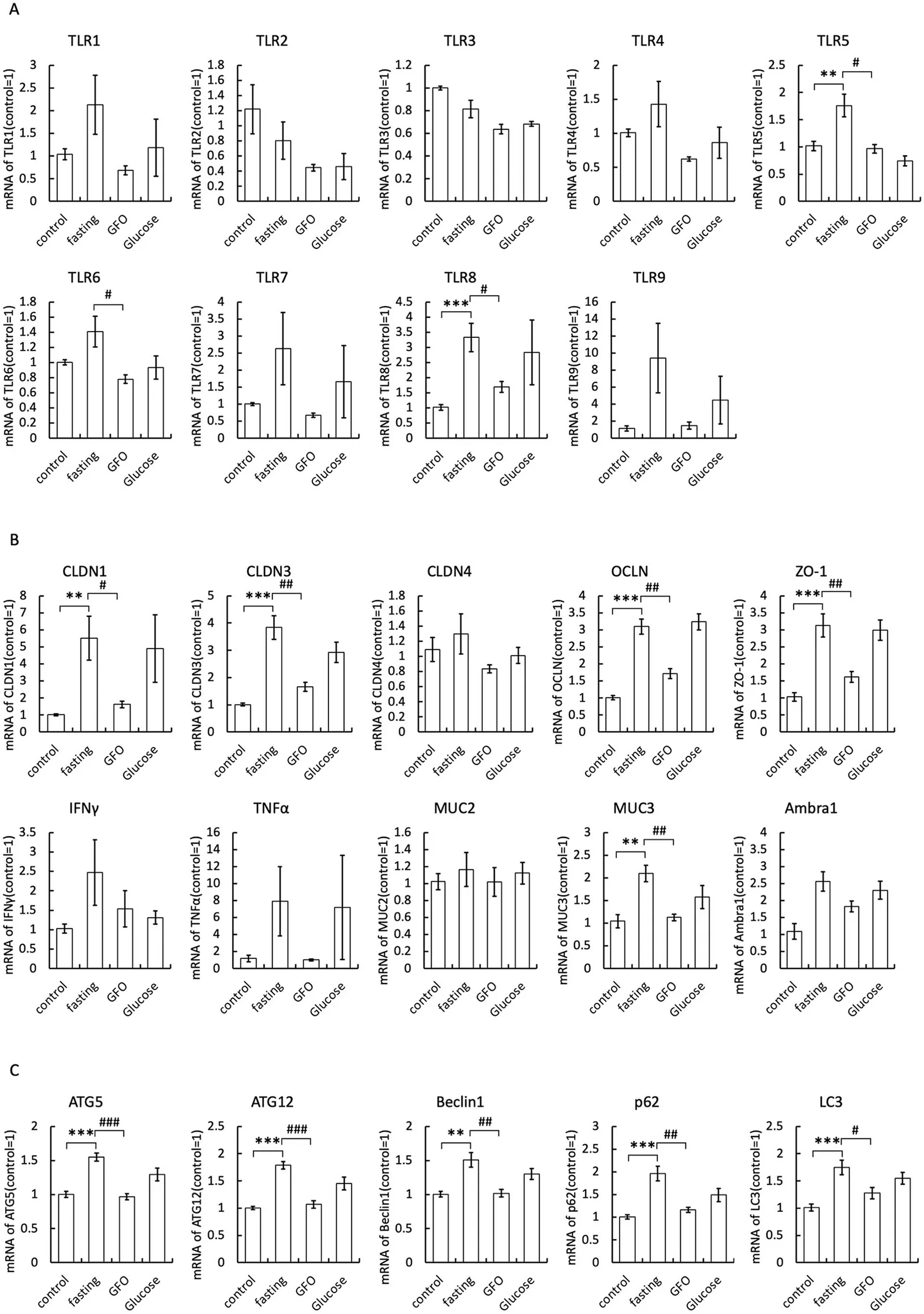
Effects of GFO administration on jejunal expression of TLR, barrier, and autophagy related genes in fasting rats. **(A)** Relative mRNA expression levels of TLR1, TLR2, TLR3, TLR4, TLR5, TLR6, TLR7, TLR8, and TLR9 in the jejunum, as determined by RT-qPCR. Expression levels were normalized to GAPDH and analyzed using the 2^−ΔΔCt^ method relative to the control group. **(B)** Relative mRNA expression levels of intestinal barrier- and immune-related genes, including CLDN1, CLDN3, CLDN4, OCLN, ZO-1, IFN-γ, TNF-α, MUC2, MUC3, and Ambra1 in the jejunum. **(C)** Relative mRNA expression levels of autophagy-related genes, including ATG5, ATG12, Beclin1, p62, and LC3 in the jejunum. All quantitative PCR (qPCR) analyses were performed using jejunal RNA samples. Gene expression levels were normalized to GAPDH as an internal control and are presented relative to the control group. Data are presented as mean ± SD. *q < 0.05, **q < 0.01, ***q < 0.001 vs. the control group. #*q* < 0.05, ##*q* < 0.01, ###*q* < 0.001 vs. the fasting group. q-values were obtained using the Benjamini–Hochberg FDR correction across the 24 genes analyzed for each comparison.

These findings indicate that fasting was associated with transcriptional changes in innate immune-, barrier-, and autophagy-related markers, whereas GFO administration attenuated several of these fasting-associated molecular changes in the jejunum.

### 3.3 GFO partially modulated fasting-induced alterations in cecal short-chain fatty acid profiles

To evaluate the effects of fasting and GFO administration on microbial metabolic activity, SCFA concentrations in cecal contents were measured immediately after euthanasia (Figure 3). Fasting significantly reduced the concentration of acetate compared with the control group, and the GFO group also showed a decreasing trend, although the difference was not statistically significant. Similarly, n-butyrate levels were significantly decreased in both the Fasting and GFO groups compared with the control group. In contrast, iso-butyrate levels were significantly increased in the Fasting group relative to the control group, whereas this increase was not observed in the GFO group. Interestingly, propionate levels were significantly elevated only in the GFO group compared with the control group. Furthermore, n-valerate concentrations were significantly reduced in both the Fasting and GFO groups compared with the control group. Iso-valerate levels were significantly increased following fasting, and no significant difference was observed between the Fasting and GFO groups. These findings indicate that prolonged fasting markedly alters cecal SCFA composition, while GFO administration partially modulates these fasting-induced metabolic changes, particularly by suppressing the increase in iso-butyrate and increasing propionate production.

**Figure 3:**
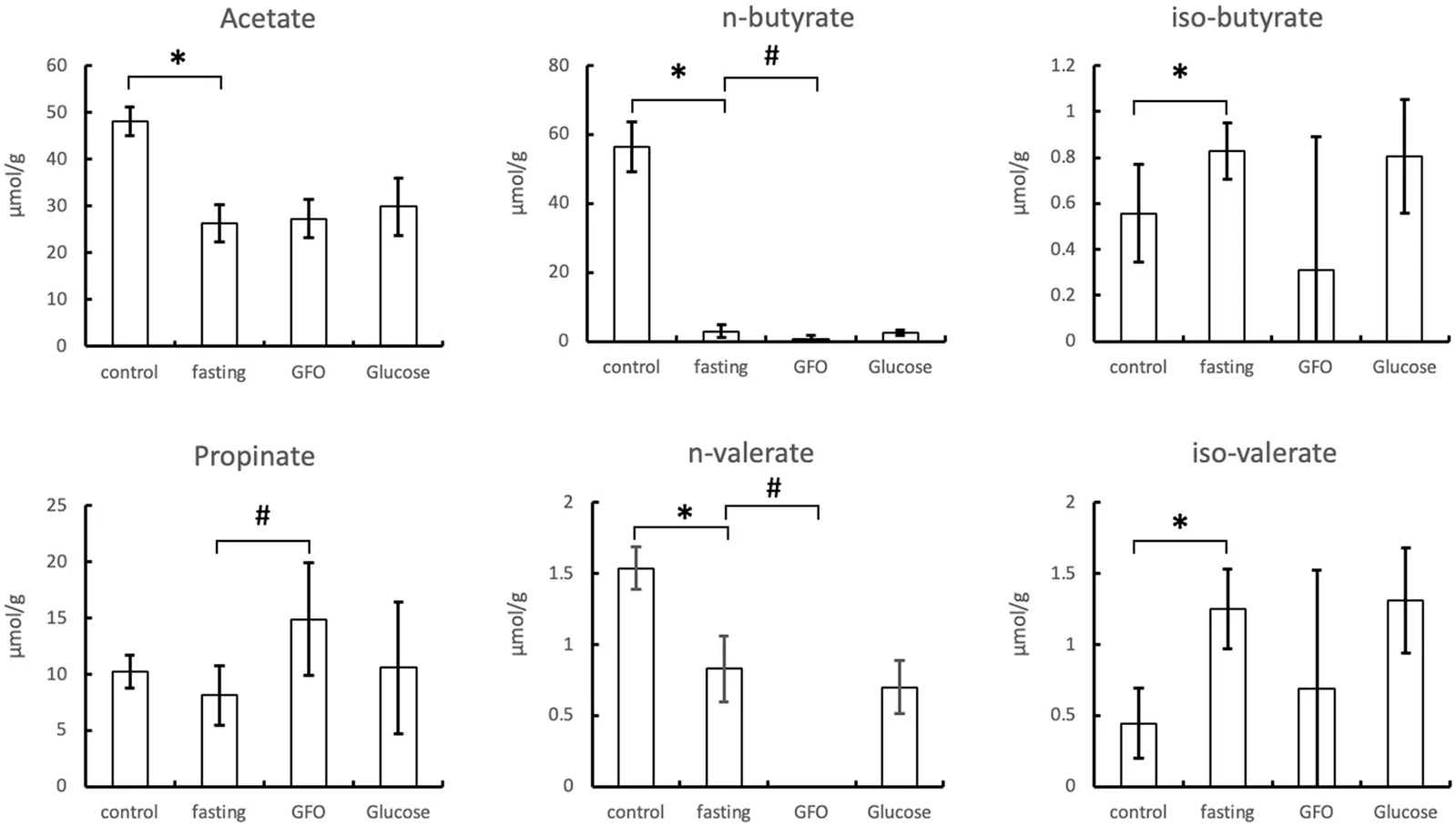
Effects of GFO administration on cecal SCFA concentrations in fasting rats. Cecal contents were collected immediately after euthanasia, and SCFA concentrations were analyzed. The concentrations of acetate, n-butyrate, iso-butyrate, propionate, n-valerate, and iso-valerate were measured in each group. Fasting significantly reduced acetate, n-butyrate, and n-valerate levels, while significantly increasing iso-butyrate and iso-valerate levels compared with the control group. GFO administration suppressed the fasting-induced increase in iso-butyrate and significantly increased propionate levels compared with the control group. Data are presented as mean ± SD (*n* = 6 per group). *p < 0.05 vs. the control group. #*p* < 0.05 vs. the fasting group.

### 3.4 Exploratory analysis of cecal microbiota composition following fasting and nutritional supplementations

To investigate the effects of fasting and GFO administration on the intestinal microbiota, 16S rDNA amplicon sequencing analysis of cecal contents was performed in the Control, Fasting, GFO, and Glucose groups (*n* = 3 per group). Principal coordinate analysis based on Bray–Curtis distances showed visually distinct clustering patterns among the four groups (Figure 4A). Control and GFO samples appeared visually separated, whereas Fasting and Glucose samples were positioned relatively close to each other. Pairwise ANOSIM showed high R values for several comparisons; however, none remained statistically significant after false discovery rate correction (all *q* ≥ 0.1344). No evidence of separation was observed between the Fasting and Glucose groups (R = −0.037, *q* = 0.611). Given the small sample size, these beta-diversity patterns were interpreted as exploratory. No significant differences in alpha diversity, as assessed by the Chao1, Shannon, and Simpson indices, were detected among the groups after false discovery rate correction (Supplementary Figure S2). Exploratory taxonomic profiling showed apparent group-dependent patterns in relative bacterial abundance (Figure 4B). Among the dominant taxa, a Lactobacillus-associated cluster appeared less abundant in the Fasting group, whereas its relative abundance was higher in the GFO group. To further characterize microbiota alterations associated with GFO treatment, several representative taxa were examined relative to the Control group (Figure 4C). Exploratory taxonomic profiling suggested that the Fasting group had higher relative abundances of sequences assigned to *Akkermansia muciniphila, Blautia, Desulfovibrio, Bilophila*, and *Mucispirillum schaedleri* than the Control group, whereas the corresponding values in the GFO group appeared closer to those observed in the Control group. In contrast, the relative abundance of sequences tentatively assigned to *Lactobacillus reuteri* appeared lower in the Fasting group, whereas the corresponding abundance in the GFO group was comparable to that in the Control group. Sequences tentatively assigned to *Bifidobacterium animalis* were detected predominantly in the GFO group and were minimally detected in the other groups. Furthermore, *Butyricicoccus pullicaecorum*, a known butyrate-producing bacterium, was detected only in the Control group and was absent in the Fasting, GFO, and Glucose groups. This abundance pattern was consistent with the reduction in n-butyrate levels; however, the present analysis does not establish a causal relationship between this taxon and SCFA production. Collectively, these exploratory findings suggest that fasting and nutritional supplementation were associated with distinct patterns of cecal microbial composition. However, the limited sample size and taxonomic resolution of 16S amplicon sequencing preclude definitive conclusions regarding group differences or individual bacterial species.

**Figure 4:**
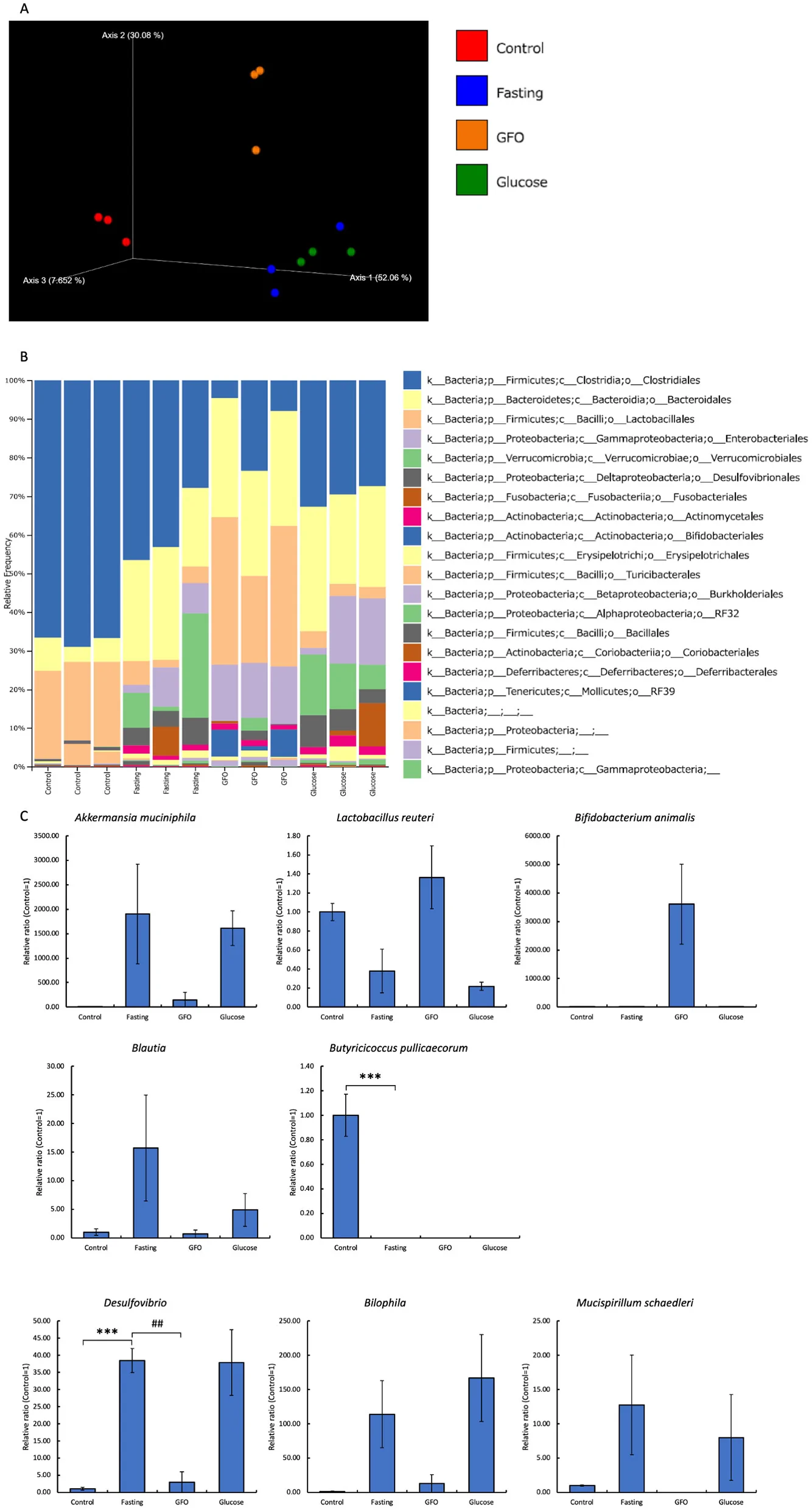
Effects of GFO administration on intestinal microbiota composition in fasting rats. **(A)** Three-dimensional Principal Coordinate Analysis (3D-PCoA) of β-diversity based on 16S rDNA amplicon sequencing of cecal microbiota. Each dot represents an individual animal. Red dots indicate the Control group, blue dots indicate the Fasting group, orange dots indicate the GFO group, and green dots indicate the Glucose group. Visually distinct clustering patterns were observed; however, no pairwise ANOSIM comparison remained statistically significant after FDR correction. **(B)** Taxonomic composition of the intestinal microbiota at the species level. Relative abundance of the top 21 bacterial taxa is shown. The vertical axis indicates relative frequency. A prominent *Lactobacillus*-associated cluster was markedly reduced in the Fasting group and enriched in the GFO group. **(C)** Relative abundance of representative bacterial species associated with intestinal barrier function and microbial metabolism, including *Akkermansia muciniphila, Lactobacillus reuteri, Bifidobacterium animalis, Blautia, Butyricicoccus pullicaecorum, Desulfovibrio, Bilophila, and Mucispirillum schaedleri*. Relative abundance was normalized to the Control group. Species-level assignments should be considered tentative because of the taxonomic resolution of 16S amplicon sequencing. Data are presented as mean ± SD (*n* = 3 per group). ***p < 0.001 vs. Control; ##*p* < 0.01 vs. Fasting.

## 4 Discussion

In the present study, we demonstrated that prolonged fasting induced substantial alterations in intestinal homeostasis, including villous atrophy, intestinal microbiota dysbiosis, altered SCFA composition, and activation of intestinal stress-related pathways involving TLRs, barrier-associated genes, and autophagy-related responses. Importantly, administration of GFO, a nutritional formulation containing glutamine, dietary fiber, and oligosaccharides, partially attenuated these fasting-associated changes. These findings suggest that preventive nutritional supplementation initiated before and continued during fasting was associated with attenuation of several fasting-related intestinal changes, accompanied by exploratory alterations in cecal microbiota composition and mucosal stress-related markers.

Preoperative fasting remains a standard perioperative management strategy intended to reduce aspiration risk and anesthesia-associated complications (1, 2). However, excessive fasting has increasingly been recognized as detrimental to metabolic and intestinal physiology. ERAS protocols now recommend minimizing prolonged fasting because nutrient deprivation induces catabolism, insulin resistance, delayed gastrointestinal recovery, and impaired postoperative outcomes (1–4). Previous studies also demonstrated that fasting impairs mucosal immunity and intestinal epithelial turnover (5, 6). Consistent with these observations, our fasting model showed marked body weight loss, reduced villous height, and decreased cecal weight, indicating significant intestinal atrophy and mucosal stress during prolonged nutrient deprivation.

The intestinal epithelium is highly dependent on luminal nutrients and microbial metabolites for maintenance of structural integrity and epithelial renewal (19, 20). Glutamine is considered a critical energy substrate for enterocytes and immune cells and has been shown to protect intestinal tissues under stress conditions (11, 12, 21). Experimental studies demonstrated that glutamine supplementation preserves epithelial tight junction organization, enhances heat shock protein expression, and reduces intestinal permeability during inflammatory or ischemic stress (11, 22, 23). In the present study, although villous height in the GFO group was not significantly different from that in either the Control or Fasting group, GFO supplementation did not significantly restore fasting-associated villous shortening. Therefore, the present morphological findings should be interpreted cautiously.

Exploratory microbiota analysis suggested group-dependent patterns in cecal microbial composition. Principal coordinate analysis showed visually distinct clustering patterns among the Control, Fasting, GFO, and Glucose groups; however, pairwise ANOSIM did not identify statistically significant differences after FDR correction. Therefore, these findings should be regarded as descriptive and hypothesis-generating rather than evidence of definitive group separation. Previous studies have demonstrated that feeding rhythms and caloric restriction strongly influence gut microbial composition and diurnal oscillation (9, 10). Fasting-associated dysbiosis has also been linked to altered microbial metabolite production and impaired intestinal immunity (5, 9).

*Lactobacillus*-associated taxa appeared less abundant in the Fasting group, whereas their relative abundance in the GFO group appeared comparable to that observed in the Control group. *Lactobacillus* species are among the best-characterized beneficial commensals and contribute to maintenance of epithelial barrier function, suppression of intestinal inflammation, production of antimicrobial metabolites, and modulation of mucosal immunity (24–26). *Lactobacillus reuteri*, detected in the present study, has been shown to induce regulatory T-cell responses and improve epithelial integrity through microbial metabolite production (27, 28). The relative abundance of sequences tentatively assigned to *Lactobacillus reuteri* in the GFO group was comparable to that in the Control group. Although this pattern coincided with attenuation of several fasting-associated intestinal changes, the present study does not establish a causal contribution of this taxon to the host responses.

Sequences tentatively assigned to *Bifidobacterium animalis* were detected predominantly in the GFO group and were minimally detected in the other groups. *Bifidobacteria* preferentially utilize oligosaccharides and dietary fibers and are major acetate-producing bacteria in the intestine (13, 29). Acetate has been shown to support epithelial barrier integrity and regulate mucosal immune responses (30). Previous clinical studies demonstrated that prebiotic supplementation promotes *Bifidobacterium* expansion and improves intestinal health (17, 18). Because the nutritional formulation contains oligosaccharides, the predominant detection of sequences tentatively assigned to *Bifidobacterium animalis* in the GFO group may be consistent with selective utilization of prebiotic substrates. Because the nutritional formulation contains oligosaccharides, this abundance pattern may be compatible with utilization of prebiotic substrates; however, species-specific and functional studies are required to confirm this interpretation.

The Fasting group showed apparently higher relative abundances of bacterial taxa associated with intestinal stress and inflammation, including *Desulfovibrio, Bilophila*, and *Mucispirillum schaedleri*. Sulfate-reducing bacteria such as *Desulfovibrio* produce hydrogen sulfide, which may impair epithelial mitochondrial respiration and barrier function (31, 32). *Bilophila* expansion has been associated with inflammatory conditions and bile acid-mediated dysbiosis (33). *Mucispirillum schaedleri* preferentially colonizes the mucus layer and expands under inflammatory conditions or altered mucus metabolism (34). The relative abundances of these taxa in the GFO group appeared closer to those observed in the Control group than to those in the Fasting group. However, the functional relevance of these abundance patterns remains to be determined.

SCFAs are key microbial metabolites that regulate intestinal epithelial metabolism, immune responses, and barrier integrity (14, 35, 36). In the present study, fasting markedly reduced acetate, n-butyrate, and n-valerate levels, while increasing iso-butyrate and iso-valerate. Reduced butyrate production may be particularly important because butyrate serves as a primary energy source for colonocytes and enhances tight junction assembly through AMP-activated protein kinase activation (37). Butyrate also suppresses intestinal inflammation and modulates regulatory T-cell differentiation (38, 39). Interestingly, *Butyricicoccus pullicaecorum*, a representative butyrate-producing bacterium with anti-inflammatory properties (40), was detected only in the Control group and disappeared under fasting conditions. This finding may partially explain the marked reduction in n-butyrate observed during fasting. Although GFO did not restore n-butyrate levels, it significantly increased propionate levels compared with the Control group. Propionate has also been reported to exert beneficial immunomodulatory and metabolic effects (41, 42), suggesting that GFO may partially compensate for fasting-associated SCFA depletion through altered microbial metabolism.

Unexpectedly, fasting induced significant upregulation of multiple barrier-associated genes, including CLDN1, CLDN3, OCLN, and ZO-1. Although intestinal injury is often associated with decreased tight junction protein expression, several studies have reported compensatory induction of barrier-related genes during epithelial stress or inflammation (43, 44). We therefore speculate that prolonged fasting triggered compensatory barrier repair responses in the jejunal mucosa. Importantly, GFO administration attenuated this transcriptional activation, suggesting reduced epithelial stress rather than simple enhancement of barrier molecule expression.

After FDR correction, fasting significantly increased TLR5 and TLR8 expression compared with the Control group, whereas GFO supplementation significantly attenuated TLR5, TLR6, and TLR8 expression compared with the Fasting group. TLR signaling plays central roles in innate immune sensing of microbial components and regulation of intestinal inflammation (45, 46). Altered exposure to microbial-associated molecular patterns is one possible explanation for the elevated TLR expression observed during fasting; however, this mechanism was not directly examined in the present study. Increased TLR4 signaling, in particular, has been implicated in intestinal barrier dysfunction and inflammatory responses (47). The attenuation of TLR-related gene expression in the GFO group occurred concurrently with exploratory changes in cecal microbiota composition; however, the present study does not establish a causal relationship between these observations.

Another important observation was the activation of autophagy-related pathways during fasting. Expression of ATG5, ATG12, Beclin1, p62, and LC3 was significantly increased under fasting conditions and attenuated by GFO administration. Autophagy is an evolutionarily conserved adaptive mechanism activated during nutrient deprivation to maintain cellular energy homeostasis (48, 49). In the intestine, basal autophagy is essential for epithelial maintenance and antimicrobial defense (50). However, excessive autophagic activation may also reflect severe cellular stress and contribute to epithelial dysfunction (51). The suppression of fasting-induced autophagy-related gene expression by GFO suggests that nutritional supplementation reduced nutrient stress at the mucosal level.

Taken together, prolonged fasting was accompanied by changes in intestinal morphology, cecal SCFA profiles, microbial composition, and intestinal stress-related markers. Combined nutritional supplementation attenuated several fasting-associated host responses and was concurrently associated with exploratory changes in microbial and metabolic profiles. However, the present study cannot determine whether the microbiota, microbial metabolites, or direct nutritional effects of the administered components were responsible for the observed intestinal phenotype. GFO administration partially mitigated these alterations through combined effects on nutrient supply, prebiotic-driven microbiota modulation, and regulation of stress-associated intestinal signaling pathways.

Several limitations should be acknowledged. First, the microbiota analysis included only three biological replicates per group and should therefore be regarded as exploratory. Although Bray–Curtis PCoA showed visually distinct clustering patterns, pairwise ANOSIM did not identify statistically significant differences after FDR correction. Species-level assignments based on 16S rRNA gene amplicon sequencing are also tentative and require confirmation using higher-resolution approaches. Second, baseline microbiota profiles were not available, and pre-existing inter-individual differences cannot be excluded. Third, the concurrent changes in microbiota composition, SCFA profiles, and intestinal molecular markers do not establish causal relationships, and functional approaches such as fecal microbiota transplantation or germ-free or microbiota-depleted models will be required to determine whether the microbiota directly mediates the observed intestinal effects. Fourth, the 15% glucose solution was included as a simple carbohydrate comparator and was not matched to GFO for caloric content, osmolality, or nutrient composition. Therefore, differences between the GFO and Glucose groups cannot be attributed specifically to individual GFO components, and effects related to differences in energy supply or physicochemical properties cannot be excluded. Fifth, supplementation was initiated before and continued during fasting, and no post-fasting treatment or recovery phase was included. Finally, although western blot analysis provided protein-level validation for ATG5 and ATG12, tight junction proteins were not evaluated at the protein level, and intestinal permeability and autophagic flux were not directly assessed. Therefore, the functional significance of the observed transcriptional changes requires further investigation.

## 5 Conclusions

Combined supplementation with glutamine, dietary fiber, and oligosaccharides attenuated several fasting-associated alterations in intestinal homeostasis in a rat model of prolonged fasting. This preventive nutritional intervention was associated with changes in gut microbiota composition and short-chain fatty acid profiles, attenuation of fasting-associated changes in TLR- and autophagy-related markers, and preservation of relative cecal weight. Because supplementation was initiated before and continued during fasting, the present findings reflect preventive rather than post-injury therapeutic effects. Further studies incorporating post-fasting treatment and recovery phases are required to determine whether this nutritional strategy can facilitate recovery from fasting-induced intestinal injury.

## Data Availability

The data supporting the findings of this study are available within the article and its Supplementary material. Raw and processed 16S rRNA gene amplicon sequencing data have been deposited in the Gene Expression Omnibus (GEO) database under accession number GSE334390. Additional data generated during this study are available from the corresponding author upon reasonable request.

## Supplementary material

**Supplementary Table S1**

Primer list.

**Supplementary Figure S1**

Protein expression of autophagy-related markers in the jejunum. **(A)** Representative western blot images of ATG12, ATG5, and β-actin in jejunal tissue from the Control, Fasting, and GFO groups. Three biological replicates per group were analyzed. **(B)** Densitometric quantification of ATG12 and ATG5 protein expression. Band intensities were quantified using ImageJ, normalized to β-actin, and expressed relative to the Control group, which was set to 1. Data are presented as mean ± SD (*n* = 3 per group). ^*^*p* < 0.05 vs. the Control group; ^#^*p* < 0.05 vs. the Fasting group.

**Supplementary Figure S2**

Alpha diversity of the cecal microbiota in the Control, Fasting, GFO, and Glucose groups. **(A)** Chao1 richness index. **(B)** Shannon diversity index. **(C)** Simpson diversity index. Alpha diversity was calculated from 16S rRNA gene amplicon sequencing data. Three biological replicates per group were analyzed (*n* = 3). Pairwise comparisons were performed using nonparametric tests, followed by Benjamini–Hochberg false discovery rate correction. No pairwise comparison remained statistically significant after FDR correction.

## References

[1] Weimann A Braga M Carli F Higashiguchi T Hubner M Klek S . ESPEN guideline: clinical nutrition in surgery. Clin Nutr. (2017) 36:623–50. doi: 10.1016/j.clnu.2017.02.013 28385477

[2] Ljungqvist O Scott M Fearon KC. Enhanced recovery after surgery: a review. JAMA Surg. (2017) 152:292–8. doi: 10.1001/jamasurg.2016.4952 28097305

[3] Braga M Gianotti L Nespoli L Radaelli G Di Carlo V. Nutritional approach in malnourished surgical patients: a prospective randomized study. Arch Surg. (2002) 137:174–80. doi: 10.1001/archsurg.137.2.174 11822956

[4] Gillis C Carli F. Promoting perioperative metabolic and nutritional care. Anesthesiology. (2015) 123:1455–72. doi: 10.1097/ALN.0000000000000795 26248016

[5] Nagai M Noguchi R Takahashi D Morikawa T Koshida K Komiyama S . Fasting-refeeding impacts immune cell dynamics and mucosal immune responses. Cell. (2019) 178:1072–87.e14. doi: 10.1016/j.cell.2019.07.047 31442401

[6] Hiesmayr M Schindler K Pernicka E Schuh C Schoeniger-Hekele A Bauer P . Decreased food intake is a risk factor for mortality in hospitalised patients: the nutritionday survey 2006. Clin Nutr. (2009) 28:484–91. doi: 10.1016/j.clnu.2009.05.013 19573957

[7] Stechmiller JK. Understanding the role of nutrition and wound healing. Nutr Clin Pract. (2010) 25:61–8. doi: 10.1177/0884533609358997 20130158

[8] Schietroma M Piccione F Carlei F Sista F Cecilia EM Amicucci G. Peritonitis from perforated peptic ulcer and immune response. J Invest Surg. (2013) 26:294–304. doi: 10.3109/08941939.2012.762073 23514054

[9] Zarrinpar A Chaix A Yooseph S Panda S. Diet and feeding pattern affect the diurnal dynamics of the gut microbiome. Cell Metab. (2014) 20:1006–17. doi: 10.1016/j.cmet.2014.11.008 PMC4255146 25470548

[10] Thaiss CA Levy M Korem T Dohnalova L Shapiro H Jaitin DA . Microbiota diurnal rhythmicity programs host transcriptome oscillations. Cell. (2016) 167:1495–510.e12. doi: 10.1016/j.cell.2016.11.003 27912059

[11] Wischmeyer PE Kahana M Wolfson R Ren H Musch MM Chang EB. Glutamine induces heat shock protein and protects against endotoxin shock in the rat. J Appl Physiol. (2001) 90:2403–10. doi: 10.1152/jappl.2001.90.6.2403 11356807

[12] Novak F Heyland DK Avenell A Drover JW Su X. Glutamine supplementation in serious illness: a systematic review of the evidence. Crit Care Med. (2002) 30:2022–9. doi: 10.1097/00003246-200209000-00011 12352035

[13] Slavin J. Fiber and prebiotics: mechanisms and health benefits. Nutrients. (2013) 5:1417–35. doi: 10.3390/nu5041417 PMC3705355 23609775

[14] Rios-Covian D Ruas-Madiedo P Margolles A Gueimonde M de Los Reyes-Gavilan CG Salazar N. Intestinal short chain fatty acids and their link with diet and human health. Front Microbiol. (2016) 7:185. doi: 10.3389/fmicb.2016.00185 PMC4756104 26925050

[15] Ohno T Mochiki E Ando H Fukasawa T Toyomasu Y Ogata K . Glutamine decreases the duration of postoperative ileus after abdominal surgery: an experimental study of conscious dogs. Dig Dis Sci. (2009) 54:1208–13. doi: 10.1007/s10620-008-0478-5 18754091

[16] Abe T Hosoi T Kawai R Uemura N Higaki E An B . Perioperative enteral supplementation with glutamine, fiber, and oligosaccharide reduces early postoperative surgical stress following esophagectomy for esophageal cancer. Esophagus. (2019) 16:63–70. doi: 10.1007/s10388-018-0630-z 30030739

[17] Zeng J Ji Y Liang B Zhang G Chen D Zhu M . The Effect of pro/synbiotics on postoperative infections in colorectal cancer patients: a systematic review and meta-analysis. Complement Ther Clin Pract. (2021) 43:101370. doi: 10.1016/j.ctcp.2021.101370 33894576

[18] Polakowski CB Kato M Preti VB Schieferdecker MEM Ligocki Campos AC. Impact of the preoperative use of synbiotics in colorectal cancer patients: a prospective, randomized, double-blind, placebo-controlled study. Nutrition. (2019) 58:40–6. doi: 10.1016/j.nut.2018.06.004 30278428

[19] Peterson LW Artis D. Intestinal epithelial cells: regulators of barrier function and immune homeostasis. Nat Rev Immunol. (2014) 14:141–53. doi: 10.1038/nri3608 24566914

[20] Turner JR. Intestinal mucosal barrier function in health and disease. Nat Rev Immunol. (2009) 9:799–809. doi: 10.1038/nri2653 19855405

[21] Kim MH Kim H. The roles of glutamine in the intestine and its implication in intestinal diseases. Int J Mol Sci. (2017) 18:1051. doi: 10.3390/ijms18051051 PMC5454963 28498331

[22] Wang B Wu G Zhou Z Dai Z Sun Y Ji Y . Glutamine and intestinal barrier function. Amino Acids. (2015) 47:2143–54. doi: 10.1007/s00726-014-1773-4 24965526

[23] Ziegler TR Evans ME Fernández-Estívariz C Jones DP. Trophic and cytoprotective nutrition for intestinal adaptation, mucosal repair, and barrier function. Annu Rev Nutr. (2003) 23:229–61. doi: 10.1146/annurev.nutr.23.011702.073036 12626687

[24] Bron PA Kleerebezem M Brummer RJ Cani PD Mercenier A MacDonald TT . Can probiotics modulate human disease by impacting intestinal barrier function? Br J Nutr. (2017) 117:93–107. doi: 10.1017/S0007114516004037 PMC5297585 28102115

[25] Plaza-Diaz J Ruiz-Ojeda FJ Gil-Campos M Gil A. Mechanisms of action of probiotics. Adv Nutr. (2019) 10:S49–66. doi: 10.1093/advances/nmy063 PMC6363529 30721959

[26] Lebeer S Vanderleyden J De Keersmaecker SC. Host interactions of probiotic bacterial surface molecules: comparison with commensals and pathogens. Nat Rev Microbiol. (2010) 8:171–84. doi: 10.1038/nrmicro2297 20157338

[27] Mu Q Tavella VJ Luo XM. Role of *Lactobacillus reuteri* in human health and diseases. Front Microbiol. (2018) 9:757. doi: 10.3389/fmicb.2018.00757 PMC5917019 29725324

[28] Atarashi K Tanoue T Oshima K Suda W Nagano Y Nishikawa H . Treg induction by a rationally selected mixture of *clostridia* strains from the human microbiota. Nature. (2013) 500:232–6. doi: 10.1038/nature12331 23842501

[29] O'Callaghan A van Sinderen D. Bifidobacteria and their role as members of the human gut microbiota. Front Microbiol. (2016) 7:925. doi: 10.3389/fmicb.2016.00925 PMC4908950 27379055

[30] Fukuda S Toh H Hase K Oshima K Nakanishi Y Yoshimura K . Bifidobacteria can protect from enteropathogenic infection through production of acetate. Nature. (2011) 469:543–7. doi: 10.1038/nature09646 21270894

[31] Loubinoux J Bronowicki JP Pereira IA Mougenel JL Faou AE. Sulfate-reducing bacteria in human feces and their association with inflammatory bowel diseases. FEMS Microbiol Ecol. (2002) 40:107–12. doi: 10.1111/j.1574-6941.2002.tb00942.x 19709217

[32] Blachier F Beaumont M Kim E. Cysteine-derived hydrogen sulfide and gut health: a matter of endogenous or bacterial origin. Curr Opin Clin Nutr Metab Care. (2019) 22:68–75. doi: 10.1097/MCO.0000000000000526 30461448

[33] Devkota S Wang Y Musch MW Leone V Fehlner-Peach H Nadimpalli A . Dietary-fat-induced taurocholic acid promotes pathobiont expansion and colitis in Il10-/- mice. Nature. (2012) 487:104–8. doi: 10.1038/nature11225 PMC3393783 22722865

[34] Herp S Brugiroux S Garzetti D Ring D Jochum LM Beutler M . *Mucispirillum schaedleri* antagonizes *Salmonella* virulence to protect mice against colitis. Cell Host Microbe. (2019) 25:681–94.e8. doi: 10.1016/j.chom.2019.03.004 31006637

[35] Koh A De Vadder F Kovatcheva-Datchary P Backhed F. From dietary fiber to host physiology: short-chain fatty acids as key bacterial metabolites. Cell. (2016) 165:1332–45. doi: 10.1016/j.cell.2016.05.041 27259147

[36] Parada Venegas D De la Fuente MK Landskron G González MJ Quera R Dijkstra G . Short chain fatty acids (SCFAs)-mediated gut epithelial and immune regulation and its relevance for inflammatory bowel diseases. Front Immunol. (2019) 10:277. doi: 10.3389/fimmu.2019.01486 PMC6421268 30915065

[37] Peng L Li ZR Green RS Holzman IR Lin J. Butyrate enhances the intestinal barrier by facilitating tight junction assembly via activation of AMP-activated protein kinase in Caco-2 cell monolayers. J Nutr. (2009) 139:1619–25. doi: 10.3945/jn.109.104638 PMC2728689 19625695

[38] Furusawa Y Obata Y Fukuda S Endo TA Nakato G Takahashi D . Commensal microbe-derived butyrate induces the differentiation of colonic regulatory T cells. Nature. (2013) 504:446–50. doi: 10.1038/nature12721 24226770

[39] Arpaia N Campbell C Fan X Dikiy S van der Veeken J deRoos P . Metabolites produced by commensal bacteria promote peripheral regulatory T-cell generation. Nature. (2013) 504:451–5. doi: 10.1038/nature12726 PMC3869884 24226773

[40] Eeckhaut V Machiels K Perrier C Romero C Maes S Flahou B . *Butyricicoccus Pullicaecorum* in inflammatory bowel disease. Gut. (2013) 62:1745–52. doi: 10.1136/gutjnl-2012-303611 23263527

[41] Hosseini E Grootaert C Verstraete W Van de Wiele T. Propionate as a health-promoting microbial metabolite in the human gut. Nutr Rev. (2011) 69:245–58. doi: 10.1111/j.1753-4887.2011.00388.x 21521227

[42] Tan J McKenzie C Potamitis M Thorburn AN Mackay CR Macia L. The role of short-chain fatty acids in health and disease. Adv Immunol. (2014) 121:91–119. doi: 10.1016/B978-0-12-800100-4.00003-9 24388214

[43] Zeissig S Burgel N Gunzel D Richter J Mankertz J Wahnschaffe U . Changes in expression and distribution of claudin 2, 5 and 8 lead to discontinuous tight junctions and barrier dysfunction in active Crohn's disease. Gut. (2007) 56:61–72. doi: 10.1136/gut.2006.094375 PMC1856677 16822808

[44] Allaire JM Crowley SM Law HT Chang SY Ko HJ Vallance BA. The intestinal epithelium: central coordinator of mucosal immunity. Trends Immunol. (2018) 39:677–96. doi: 10.1016/j.it.2018.04.002 29716793

[45] Akira S Takeda K. Toll-like receptor signalling. Nat Rev Immunol. (2004) 4:499–511. doi: 10.1038/nri1391 15229469

[46] Abreu MT. Toll-like receptor signalling in the intestinal epithelium: how bacterial recognition shapes intestinal function. Nat Rev Immunol. (2010) 10:131–44. doi: 10.1038/nri2707 20098461

[47] Cario E. Toll-like receptors in inflammatory bowel diseases: a decade later. Inflamm Bowel Dis. (2010) 16:1583–97. doi: 10.1002/ibd.21282 PMC2958454 20803699

[48] Mizushima N Komatsu M. Autophagy: renovation of cells and tissues. Cell. (2011) 147:728–41. doi: 10.1016/j.cell.2011.10.026 22078875

[49] Levine B Kroemer G. Autophagy in the pathogenesis of disease. Cell. (2008) 132:27–42. doi: 10.1016/j.cell.2007.12.018 PMC2696814 18191218

[50] Deretic V Levine B. Autophagy balances inflammation in innate immunity. Autophagy. (2018) 14:243–51. doi: 10.1080/15548627.2017.1402992 PMC5902214 29165043

[51] Cadwell K. Crosstalk between autophagy and inflammatory signalling pathways: balancing defence and homeostasis. Nat Rev Immunol. (2016) 16:661–75. doi: 10.1038/nri.2016.100 PMC5343289 27694913

